# A Perovskite Material Screening and Performance Study Based on Asymmetric Convolutional Blocks

**DOI:** 10.3390/ma17153741

**Published:** 2024-07-28

**Authors:** Shumin Ji, Yujie Zhang, Yanyan Huang, Zhongwei Yu, Yong Zhou, Xiaogang Lin

**Affiliations:** 1 School of Physics and Technology, Nantong University, Nantong 226001, China; aassdehj123@163.com (S.J.); zyj229583@163.com (Y.Z.); yyhuang@ntu.edu.cn (Y.H.); 2 Key Laboratory of Optoelectronic Technology and System of Ministry of Education, College of Optoelectronic Engineering, Chongqing University, Chongqing 400044, China; xglin@cqu.edu.cn

**Keywords:** asymmetric convolution, perovskite, residual networks, prediction models

## Abstract

This study introduces an innovative method for identifying high-efficiency perovskite materials using an asymmetric convolution block (ACB). Our approach involves preprocessing extensive data on perovskite oxide materials and developing a precise predictive model. This system is designed to accurately predict key properties such as band gap and stability, thereby eliminating the reliance on traditional feature importance filtering. It exhibited outstanding performance, achieving an accuracy of 96.8% and a recall of 0.998 in classification tasks, and a coefficient of determination (R^2^) value of 0.993 with a mean squared error (MSE) of 0.004 in regression tasks. Notably, DyCoO_3_ and YVO_3_ were identified as promising candidates for photovoltaic applications due to their optimal band gaps. This efficient and precise method significantly advances the development of advanced materials for solar cells, providing a robust framework for rapid material screening.

## 1. Introduction

Due to their relatively high power conversion efficiency and low production cost, perovskite solar cells (PSCs) have attracted considerable research interest [1]. In the last few years, the power conversion efficiency of PSCs has risen from 3.8% to 25% [2]. This advancement outweighs the capabilities of traditional silicon-based solar cells [3]. The multitude of benefits associated with perovskite solar cells originates from their exceptional light absorption capabilities and superior charge transfer properties. Perovskite materials are highly valued for their excellent light absorption and charge transfer properties, making them ideal for solar cells and various optoelectronic devices [4]. By altering their morphology and dimensions, perovskites can form one-dimensional, two-dimensional, or three-dimensional structures, which hold great promise for environmental and energy challenges. Additionally, they are gaining attention for water pollution adsorption and photocatalysis, with significant potential in these areas [5].

The performance of perovskite materials is intricately linked to the stability and regularity of their crystal structure. Variations in performance are attributed to differences in the types and stoichiometry of chemical elements situated at distinct positions within the crystal structure. Perovskite materials commonly exhibit various structural types such as ABX_3_, double A-site, and double B-site perovskites [6]. The ternary ABX_3_ structure showcases a distinct and organized distribution of ions, with a relatively straightforward elemental stoichiometry, making it the most prevalent perovskite compound [7,8].

Perovskite materials have become a significant area of focus in solar cell research due to their remarkable performance [9]. Scholars are particularly captivated by their characteristics and structural integrity. Traditional methodologies often rely on experimental validation and iterative trial-and-error processes. The performance of materials is rigorously tested through repeated experiments until the properties of simulated materials align with the predefined targets. This research approach demands the utilization of limited resources and involves complex experimental protocols, presenting substantial challenges in terms of time, labor, and costs [10]. In recent years, the proliferation of material data has enabled the application of machine-learning techniques to rapidly predict novel and uncharted compounds. This capability is derived from machine learning’s ability to reorganize existing knowledge frameworks and reveal hidden relationships. Li et al. (2019) introduced a method based on transfer-learning strategies to evaluate the stability of these compounds by predicting the formation energy of stable perovskite materials using machine learning, transfer learning, and convolutional neural networks. Among the 764 potential materials screened, 98 stable materials were confirmed through density functional theory (DFT) calculations, identified as key candidates for further study. However, this method is limited by the small size of the screened dataset and its moderate performance [11]. In 2021, Gao et al. proposed an innovative approach that combined machine-learning techniques and DFT calculations for high-throughput screening of 5796 inorganic double perovskite compounds. This study was the first to apply the extreme gradient-boosting regression (XGBR) algorithm in the development of machine-learning models for perovskite materials, demonstrating lower MSE compared to artificial neural networks (ANNs) and support-vector regression algorithms. This model identified two new inorganic double perovskite compounds [12]. In 2022, the elastic net regression (ENR) model accurately predicted metal halide perovskites’ properties, showing a high correlation of 0.98 with DFT calculations [13]. Despite their accuracy, light gradient-boosting machine regressor (LGBMR) and extreme gradient-boosting classifier (XGBC) have limited generalization across datasets. In 2024, they excelled in predicting double perovskite properties through classification and regression [14]. However, challenges remain in unifying these tasks within a single model, and there is still room for improvement in accuracy.

In this study, we introduce the asymmetric convolutional residual network (ACRNet), a deep-learning hybrid model for both classification and regression across datasets, using various-sized kernels to enhance feature extraction and reduce computational load [15]. The residual block (ResBlock) incorporates residual connections, effectively mitigating the vanishing-gradient problem in deep networks [16]. The combination of these two modules not only balances model complexity and computational efficiency but also enhances the model’s feature representation capability.

ACRNet, leveraging the efficient computational power of deep learning, significantly reduces computation time and resource consumption compared to traditional DFT calculations [17]. Additionally, our model outperforms previously mentioned machine-learning models such as XGBR, ENR, LGBMR, and XGBC. ACRNet performs both classification and regression, offering comprehensive results and enhanced flexibility for various predictive needs, increasing its practical value.

## 2. Materials and Methods

### 2.1. Dataset

A crucial requirement for predicting the stability of perovskite structures through deep-learning models is the acquisition of a large volume of high-quality data with well-defined crystal structures. Traditional computational methods, such as DFT [18], along with resources like inorganic crystal structure repositories and the materials project [19], serve as the primary sources of data for materials research. Despite the availability of these sources, collecting extensive datasets remains a significant challenge, constrained by time and financial costs. Without sufficient data, the reliability of machine-learning model predictions cannot be ensured. To address this, we streamlined and refined the dataset on solar thermal chemical hydrogen initially proposed by Zhai et al. [20]. We retained only features related to thermal stability, ultimately curating approximately 1400 data points. Our method queries the Inorganic Crystal Structure Database for non-perovskite ABO_3_ structures, using additional data like space groups to ensure accuracy, supplemented by literature reviews. For example, FeTiO_3_ is ilmenite [21].

To enhance the reliability of our analysis, we rigorously cleaned the raw data. This process addresses two major issues. Firstly, the repetition of data queries often leads to duplications, which can clutter the dataset. Secondly, it is crucial to remove data entries containing radioactive elements and rare-earth elements, owing to environmental and economic concerns. Given the inefficiencies associated with manual screening, we have adopted code-based techniques to automate the filtering and elimination of specific data types. This automated approach significantly reduces the time spent on data processing and enhances the efficiency and accuracy of our dataset management.

The database we utilize classifies a material as a perovskite, which serves as a label for the predictions made by our mixed convolutional model. For the regression prediction capabilities of the model, we employ the built-in dataset from Matminer, enriching it with additional descriptors to create a new dataset. Matminer is a Python library specifically designed for the materials science and computational materials science fields [22]. This system provides interfaces that enable users to access various material databases for both experimental and computational data. Additionally, Matminer houses a repository that includes 40 datasets from machine-learning studies and high-throughput computational research on material properties, all of which have been published and peer-reviewed. One notable dataset within Matminer is “Wolverton_oxides”, which comprises 4914 perovskite oxides detailing composition, lattice constants, formation energies, and vacancy formation energies. All listed perovskites adhere to the ABO_3_ chemical formula. This dataset is derived from research conducted by Emery and Wolverton [23]. Within the dataset, the atoms located at the A and B sites of the perovskite and the local distortion crystal structures that exhibit the lowest energy among considered distortions are non-numeric data. To facilitate more efficient model training, these three features have been excluded from the dataset.

### 2.2. Feature Selection

The descriptors for perovskites are initially delineated by consolidating those frequently employed in the existing literature. Key descriptors include the Goldschmidt tolerance factor (t) [24], the octahedral factor (μ) [25], and the new tolerance factor (τ) [26], which are widely used in studies of perovskite materials. The ionic radius and oxidation state of elements A and B are also integral to the perovskite signature, as these factors are closely interrelated with the aforementioned descriptors. Additionally, it is advisable to incorporate the ratio of A-position cations to oxygen ions (r_A_/r_O_), a parameter derived from the octahedral factor, into the descriptor set. Given that our dataset focuses on doped fractional perovskites [27], it is crucial to consider the effects of multiple doped ions at both the A and B sites of the perovskites. Besides the 12 fundamental characteristics, 9 additional traits have been included to enrich the descriptor set and enhance predictive modeling accuracy.

Understanding the relationships between the structural properties of perovskite materials clarifies their performance link to microstructures, aiding material design. The crystal structure affects their electronic, optical, and mechanical features; lattice parameters and symmetry influence optoelectronic properties; while doping modulates these aspects and introduces new energy levels.

The data features for the built-in dataset in the Matminer library are detailed in the official documentation for Matminer version 0.9.0. The Wolverton_oxides dataset comprises 16 features, of which 13 have been selected as descriptors for our study. For the exact definitions of these descriptors, please refer to Table S1.

### 2.3. Model

We propose a hybrid neural network with ACBs and ResBlocks, surpassing ANN, XGBR, and ENR in perovskite prediction. ResBlocks extract deep features, preventing gradient vanishing, while ACBs enhance expressiveness and simplify complexity. Using other methods like ANN would lower accuracy and lose features, diminishing performance.

**Figure 1:**
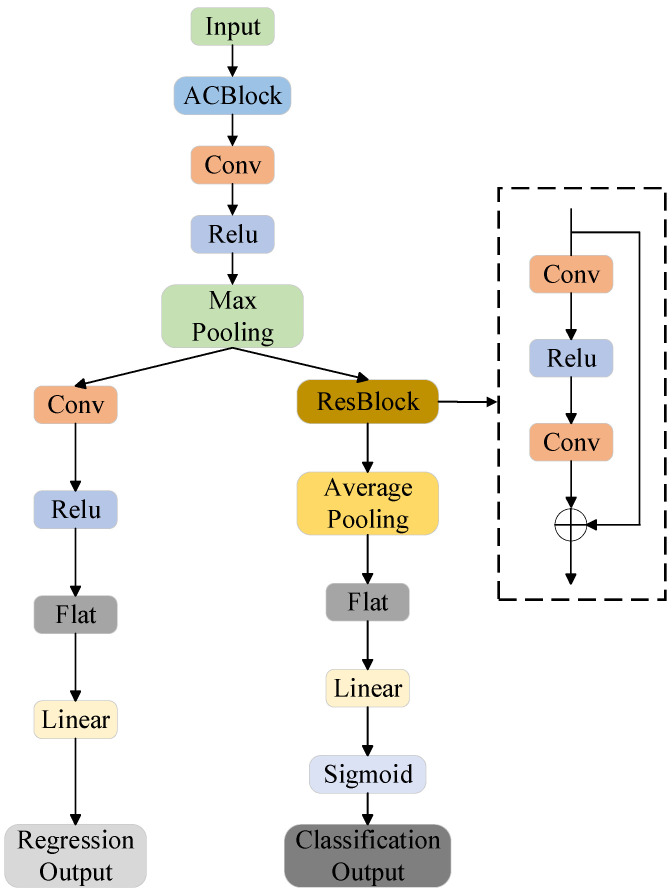
The overall framework of the model is depicted in the diagram. The left branch performs regression tasks, while the right branch handles classification functions. Within this framework, the ResBlock structure is represented by the dashed line on the far right of the diagram.

Figure 1 shows a neural-network model implemented in PyTorch, an open-source framework using artificial intelligence, known for its dynamic graph and easy application programming interface (API) [28]. The version of PyTorch is 1.7.1. Our model utilizes an i5-7300HQ central processing unit (CPU) and a 1050 graphics processing unit (GPU). The python version employed is 3.7.

The network is characterized by an asymmetric convolution module, a standard convolution module, and a residual module. The input to the network is a 4 × 5 matrix. The first layer utilizes an asymmetric convolution network [15], which is designed to extract original features from irregular two-dimensional arrays and transform them into uniformly dimensioned array data. The second layer of the network employs a 3 × 3 convolution kernel with 32 channels, followed by a rectified linear unit (ReLU) activation function and a subsequent maximum pooling layer. This convolutional layer enhances feature extraction by identifying maximum local values, highlighting key features. The third layer offers pathways for regression with additional layers, and for classification with residual modules and pooling. Due to variations in input and output sizes between these convolutional layers, data padding is necessary to maintain dimension consistency. Incorporation of a ReLU activation layer is crucial across both pathways to introduce nonlinearity and prevent vanishing gradients, thereby enhancing the model’s learning efficacy and stability.

Prior to being fed into the model, the data undergo standardization. Initially, the data are transformed into a two-dimensional array with 4 rows and 4 above columns using ACBs. These reshaped data are then used to calculate the mean and standard deviation. Following this, the data are normalized, ensuring that each feature contributes evenly to the analysis, thus enhancing the predictive performance of the model.

For the first time, ACBs are being utilized in a model designed to predict material stability and properties. This integration significantly enhances the compatibility between the model and the material data. Conventionally, it is necessary to first filter out certain material characteristics to predict the material data, which can result in the loss of critical original information, thereby potentially diminishing the predictive performance of the model. The ACB effectively resolves this challenge by incorporating comprehensive feature information from the material data during the training phase. This approach ensures that all pertinent data are utilized, maintaining the integrity and accuracy of the predictions made by the model.

In general, when considering a two-dimensional input image *I* and an asymmetric convolutional kernel *K*, the computation of the ACB can be represented by the following formula [15]:

(1) $$O\left[i,j\right]=\sum_{m}\sum_{n}I\left[i+m,j+n\right]\cdot K\left[m,n\right]$$

Within this context: *O* [*i*, *j*] represents the value of the position within the output array (*i*, *j*). *I* [*i+m*, *j+n*] represents the value of the position within the input array [*i+m*, *j+n*]. The weight of the convolutional kernel is denoted by *K* [*m*, *n*], while *m* and *n* represent the row and column indexes of the kernel, respectively.

The defining characteristic of ACB is the imbalance in the convolution kernel’s weights in the horizontal and vertical directions. This distinctive feature significantly enhances the model’s adaptability to various input data dimensions. By leveraging this asymmetry, the model can more effectively capture and retain the original data features, ensuring that critical information is preserved throughout the learning process, thereby enhancing the overall accuracy and efficiency of the model.

Adding ACB to ACRNet provides significant advantages over other common machine-learning models, such as support-vector machine (SVM), random-forest regression (RFR), gradient-boosting regression (GBR), XGBC, and XGBR, in terms of enhanced feature extraction and reduced computational load. SVM optimizes a hyperplane for classification, excelling in high-dimensional data and small samples, outperforming ensemble methods like RFR and XGBR [29]. RFR uses random trees for applications like loan risk and disease prediction [30]. GBR integrates weak learners for enhanced prediction in fields like finance and bioinformatics [31]. XGBC excels in classification with fast computation and strong generalization, used in finance, sales forecasting, and biomedical analysis [32].

The pivotal component of the model architecture is the residual block. The fundamental concept behind residual blocks is to facilitate the learning of identity mappings within the network by incorporating residual connections between layers [33], which enable certain layers to bypass others directly. The fundamental structure of the ResBlock is outlined as follows [34]: The input variable *x* undergoes transformation through the convolutional layer denoted as

(2) $$F\left(x\right)=W_{2}\left(r\left(W_{1}\left(x\right)\right)\right)$$

where *W*_1_ and *W*_2_ represent the weight parameters of the convolutional kernel, and *r* denotes the activation function. Add the transformed feature *F*(*x*) to the input to obtain the following [34]:

(3) $$y=F\left(x\right)+x$$

The residual structure allows the network to bypass the convolutional layer, thereby eliminating the need for the network to learn the entire mapping. This method also alleviates the problem of vanishing network gradients. Simultaneously, the input is transmitted to the output through a jump connection, helping to preserve the original features. The incorporation of the residual module significantly enhances the model’s predictive performance.

Our model uses a residual network for better optimization, and simple classifiers decrease accuracy and limit performance. ACBs enhance capability and reduce complexity, increasing predictive performance. In the subsequent research, we will compare the performance of our model with other models such as SVM and RFR. In classification tasks, evaluation metrics include accuracy, F1 score, recall, and area under the curve (AUC) value. For regression tasks, we will use multiple error metrics such as MSE, root mean squared error (RMSE), mean absolute error (MAE), and R^2^ for performance assessment. The F1 score combines precision and recall, while AUC evaluates the overall performance of classifiers. MSE and RMSE measure prediction errors, MAE reflects prediction accuracy, and R^2^ assesses model fit. Specifically, RMSE is the square root of MSE. The specific formulas are shown below [35]:

(4) $$F1\;\mathrm{score}=\frac{2\times \frac{\mathrm{TP}}{\mathrm{TP}+\mathrm{FN}}\times \mathrm{recall}}{\frac{\mathrm{TP}}{\mathrm{TP}+\mathrm{FN}}+\mathrm{recall}}$$

(5) $$\mathrm{RMSE}=\sqrt{\frac{\sum_{1}^{N}{\left(Y_{1p}-Y_{2p}\right)}^{2}}{N}}$$

(6) $$\mathrm{MAE}=\frac{\sum_{1}^{N}\left|Y_{1p}-Y_{2p}\right|}{N}$$

(7) $$R^{2}=1-\frac{\sum_{1}^{N}{\left(Y_{1p}-Y_{2p}\right)}^{2}}{\sum_{1}^{N}{\left(Y_{1p}-Y_{3p}\right)}^{2}}$$

Here, *Y*_1*p*_ and *Y*_2*p*_ represent the actual value and the predicted value of the *p*th instance in the test dataset, respectively, and *Y*_3*p*_ represents the mean actual value of all *N* instances [35].

Furthermore, we use kernel density estimation (KDE) to show the probability density function curve, based on independent observations from an unknown distribution [36]. Consequently, these observations can be regarded as a sample from an unknown distribution. In a mathematical context, when considering a set of observations denoted as [36]

(8) $$\left\{x_{1},x_{2},\ldots ,x_{n}\right\},$$

the kernel density estimate is represented as [36]

(9) $$\hat{f}\left(x\right)=\frac{1}{nh}\sum_{i=1}^{n}K\left(\frac{x-x_{i}}{h}\right)$$

where *K* represents the kernel function, and *h* denotes the bandwidth parameter.

## 3. Results and Discussion

Our model can perform both classification and regression tasks within the same framework (see Section 3.1 and Section 3.2). Therefore, we need to test the predictive performance of the system for both types of tasks. As the framework includes residual modules, we need to tune the number of residual layers to achieve optimal performance. Subsequently, we will compare the optimal performance of our method with other common machine-learning methods to demonstrate its superior performance in both tasks. Based on this, we will proceed with the prediction of perovskite materials.

### 3.1. Perovskite Stability Prediction

**Figure 2:**
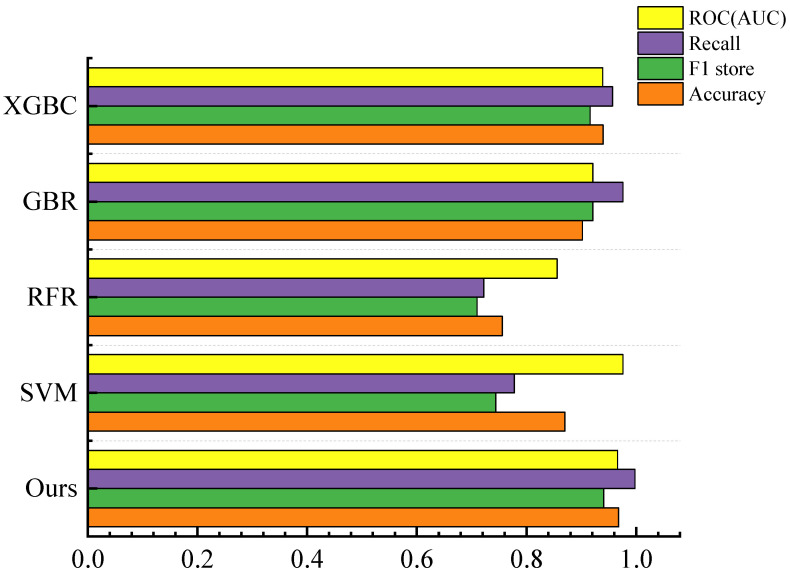
Evaluation metrics for models and other machine-learning models.

Utilizing PyTorch, our study successfully achieves the classification and prediction of perovskite oxide stability. The performance of all classification models is rigorously assessed using metrics such as accuracy, F1 score, recall, and AUC. In our analysis, we also compare our models with established classification approaches, including SVM [29], RFR [30], GBR [31], and XGBC [32]. The evaluation of these classifiers is based on a randomly generated training set comprising 20% of the data. Detailed results of these classifications are presented in Figure 2.

As illustrated in Figure 2, significant variations in the evaluation results are observed among different models. The RFR classifier recorded the lowest average values, with an accuracy of 0.756, F1 score of 0.707, recall of 0.721, and AUC score of 0.856. In stark contrast, our model demonstrated superior performance, achieving the highest scores across all metrics. Compared to the RFR classifier, our model exhibited substantial improvements, with enhancements in accuracy and F1 score of approximately 0.2, and improvements in recall and AUC of around 0.1.

### 3.2. Perovskite Band Gap, Formation Energy, and Convex Hull Energy Prediction

**Figure 3:**
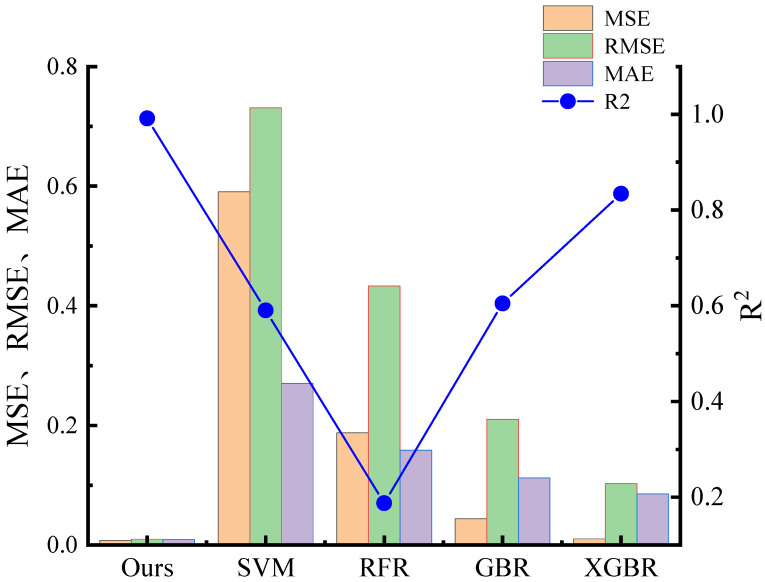
Our model is compared with the evaluation metrics of SVM, RFR, GBR, and XGBR. The three columns represent the values of MSE, RMSE, and MAE, respectively, corresponding to the left Y-axis, while the line represents the value of R^2^, corresponding to the right Y-axis.

The model’s classification predictions effectively discern the stability of perovskite oxides. Additionally, regression analysis provides a deeper understanding of perovskite materials, revealing intricate details such as formation energy, predictive energy, and convex hull energy. By adjusting the features considered in the dataset, researchers can tailor the model to accurately identify the most suitable perovskite material. Convolutional networks are utilized for regression predictions. Our model was also compared with other classifiers including SVM, RFR, GBR, and XGBR. The evaluation was based on an average of several key metrics: MSE, RMSE, MAE, and R^2^. The regression performance of all models is illustrated in Figure 3.

The SVM classifier is commonly used for classification predictions and reports an MSE of 0.591, an RMSE of 0.731, and an MAE of 0.270, which are higher than those observed in alternative models. In contrast, the RMSE for the RFR, GBR, and XGBR classifiers was recorded as below 0.5, while the MAE remained under 0.3, indicating superior performance in regression predictions. Our model outperformed these classifiers significantly, with notably lower MSE, RMSE, and MAE values of 0.004, 0.01, and 0.010, respectively. Additionally, it achieved an impressive R^2^ of 0.993, demonstrating a substantial predictive advantage. Among the other classifiers, only the GBR and XGBR achieved R^2^ values above 0.9, at 0.903 and 0.954, respectively.

**Figure 4:**
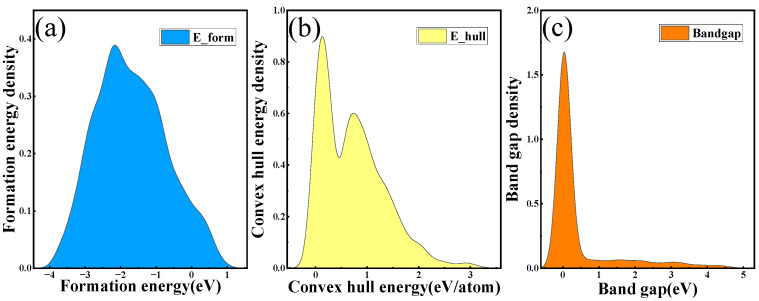
The model employed to predict features in the KDE graph is structured as follows: (**a**) represents the formation energy, (**b**) depicts the convex hull energy, and (**c**) illustrates the band gap.

We have produced KDE plots to illustrate the distribution of formation energy, convex hull energy, and band gap, as shown in Figure 4. The KDE analysis indicates that the estimated formation energy for perovskite oxides in our dataset predominantly ranges from 0.2 to 0.8. Meanwhile, the convex hull energy is expected to be within 10 to 15, and the band gap is projected to range approximately between 4 and 5.8. Figure 4 provides a clear and intuitive depiction of the feature predictions for the perovskite dataset. This visual representation assists researchers in designing experiments that are well-aligned with the intrinsic properties of perovskite materials.

**Figure 5:**
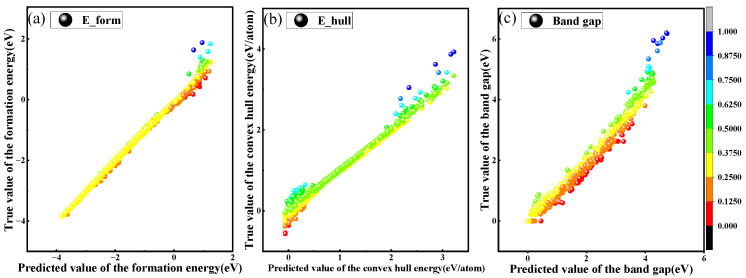
Scatter plot of model predicted values, where colors represent the difference between the actual values and the predicted values. (**a**) The graph represents the formation energy; (**b**) the graph represents the convex hull energy; (**c**) the graph represents the band gap.

Scatter plots illustrating formation energy, convex hull energy, and band gap are used to effectively highlight the discrepancies between the predicted and actual values, as shown in Figure 5. The comparison reveals that the convex hull energy predictions align most accurately with the regression line, indicating a high level of predictive accuracy. However, the band gap predictions show significant deviation from the regression line, particularly at a notable outlier around 5.5–6, suggesting a reduced predictive capability in this range. Additionally, the predictive accuracy for values between 1.5 and 3.0 diminishes, leading to increased dispersion around the regression line, which further indicates areas where the model’s performance could be improved.

### 3.3. Model Parameter Optimization

The performance of the model is impacted by the number of layers in the residual module, but more layers do not necessarily equate to improved performance [37]. As demonstrated in Table 1, the relationship between the number of layers and the model’s accuracy varies. With no layers in the residual module, the model achieves a classification prediction accuracy of 0.884. However, the accuracy peaks at 0.968 with two layers, suggesting an optimal level of complexity for this model configuration. Beyond this point, an increase in the number of layers leads to a decline in prediction performance, a trend that can be attributed to overfitting issues that emerge when the model is overly trained on the dataset. Careful adjustment and optimization of the number of layers are crucial for maximizing performance across different datasets. Through experiments, we found that the optimal layer configuration can significantly enhance the accuracy and efficiency of the model.

### 3.4. Comparison of Model Metrics

We compared our method with those reported in the recent literature. The classification models include XGBC [14], gradient-boosting classifier (GBC) [38], and tree-based pipeline optimization tool classifier (TPOTC) [38]. The regression models include light gradient-boosting machine (LightGBM) [39], catboost regressor (CBR) [40], and polynomial regression network (PRN) [41]. Table 2 and Table 3 present the performance metrics comparison for classification and regression tasks, respectively. Table 2 shows our model excels in classification, improving all metrics except the F1 score compared to XGBC. Table 3 confirms our model significantly surpasses the recent literature, validating its effectiveness and potential in enhancing classification tasks.

### 3.5. Screening of Solar-Cell Materials

In our latest study, a deep-learning hybrid model employing asymmetric convolutional residual networks is utilized to predict data features for perovskite oxides, focusing specifically on forecasting convex hull energies and band gaps. This predictive modeling aids in identifying perovskites with favorable photovoltaic properties based on their band gap measurements. The refined dataset encompasses 1785 compounds, with 19 features including oxidation states, formation energies, and atomic radii at A and B sites, all calculated using first-principles density functional theory.

The model’s predictions classify perovskite oxides with convex hull energies below 36 meV/atom as stable and those below 70 meV/atom as metastable. Importantly, band gaps predicted to be between 1.1 eV and 1.5 eV indicate high potential for solar-cell efficiency.

**Figure 6:**
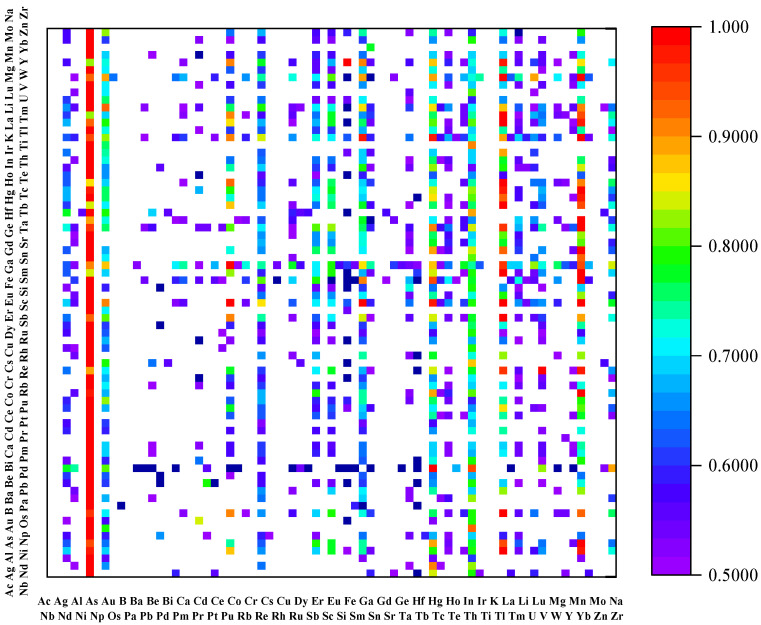
Probability heatmap generated by the model based on the predicted convex hull energies, displaying materials in the dataset with a stability or metastability probability greater than 50%. The depth of the colors indicates the probability of forming perovskite: the deeper the yellow, the higher the probability; the deeper the blue, the lower the probability.

Table 4 provides a detailed outline of the predicted results for convex hull energies and band gaps, based on which we identified 30 materials as potential stable perovskites. The stability of 12 of these materials has been validated through the literature references. Figure 6 illustrates a probability heatmap of the predicted convex hull energies, showing materials with a stability or metastability probability greater than 50%; deeper shades of yellow represent higher probabilities, while deeper blue signifies lower probabilities. For instance, according to reference [42], the direct band gap of NaPuO_3_ was measured as 1.1 eV, which is extremely close to our predicted value of 1.1739 eV. Additionally, reference [43] reports that CaPbO_3_ exhibits a direct band gap of 0.94 eV, which compares reasonably well with our prediction of 1.1269 eV. Given their optimal band gaps for photovoltaic applications, materials like DyCoO_3_ and YVO_3_ are highlighted as promising candidates for solar-cell materials.

## 4. Conclusions

We propose a deep-learning hybrid model based on an ACRNet to predict the stability and properties of perovskite materials. This model excels at handling various material datasets and distinguishing features, thereby facilitating the accurate identification of specific materials. The detailed structure and performance of the model are as follows:

(1) Feature extraction and model structure: Initially, an ACB is employed to retain as much original feature information as possible, enhancing the model’s flexibility and generalization capabilities. Additionally, the use of ResBlock allows for skipping specific convolutional layers, thereby reducing the model’s complexity and computational demands and improving its suitability for large-scale material data predictions.
(2) Classification task performance: In classification tasks, our model has demonstrated exceptional predictive performance, achieving an accuracy of 0.968 and an AUC of 0.965.
(3) Regression task performance: In regression tasks, it has achieved an R^2^ value of 0.993, with lower MSE, RMSE, and MAE values compared to conventional machine-learning models.
(4) Prediction of perovskite oxides: In the latest dataset predictions, based on convex hull energies and band gaps, materials such as DyCoO_3_ and YVO_3_ have been identified as promising candidates for solar-cell applications. This showcases the model’s extensive potential in predicting material stability, properties, and in effectively screening materials.

The relevance of our work lies in its ability to provide materials scientists with a comprehensive tool to quickly and accurately predict material properties, which is crucial for the development of new materials. The ACRNet model boosts predictive accuracy and efficiency, serving materials scientists in developing new materials and enhancing battery, semiconductor, and catalyst functionalities. Future research may expand datasets, integrate real-time predictions, and improve generalization and accuracy.

## Figures and Tables

**Table 1:** The impact of residual layer on model accuracy and R^2^.

Number of Layers	Accuracy	R^2^
0	0.884	0.987
2	0.968	0.993
4	0.942	0.992

**Table 2:** Comparison of metrics for classification tasks.

Model	Accuracy	Recall	F1 Score	AUC
XGBC [14]	0.950	0.960	0.940	0.950
GBC [38]	0.862	0.880	0.882	0.930
TPOTC [38]	0.838	0.851	0.861	0.933
Ours	0.968	0.998	0.941	0.966

**Table 3:** Comparison of metrics for regression tasks.

Model	MSE	RMSE	MAE	R^2^
LightGBM [39]	0.545	0.738	0.430	0.973
CBR [40]	0.014	0.120	0.083	0.790
PRN [41]	0.176	0.420	0.074	0.962
Ours	0.001	0.010	0.009	0.991

**Table 4:** Model-predicted convex hull energy and band gap.

ABO_3_	E_hull_ [eV/atom]	Band Gap [eV]	ABO_3_	E_hull_ [eV/atom]	Band Gap [eV]
ErVO_3_	−0.0241	1.3734	GdVO_3_	−0.0408	1.4539
CeFeO_3_	−0.0555	0.9472	CaPbO_3_	0.0347	1.1269
SmFeO_3_	−0.0316	1.1743	LuFeO_3_	0.0021	1.2930
YFeO_3_	−0.0334	1.1794	AgNbO_3_	0.0430	1.2360
GdFeO_3_	−0.0273	1.0481	BiFeO_3_	0.0115	1.1050
TbFeO_3_	−0.0203	1.1870	LaTiO_3_	0.0289	1.1780
PrCoO_3_	−0.0019	1.0769	PrVO_3_	−0.0561	1.4650
DyFeO_3_	−0.0353	1.0784	PbPuO_3_	−0.0333	1.2032
NaPuO_3_	−0.0555	1.1739	TmVO_3_	−0.0126	1.3021
KPuO_3_	−0.0833	1.1600	HoCoO_3_	0.0299	1.4519
HoFeO_3_	−0.0271	1.1136	DyCoO_3_	0.0171	1.2701
YbPbO_3_	0.0295	1.2060	HgPuO_3_	−0.0201	1.1873
AcVO_3_	−0.0665	1.2351	HgHfO_3_	0.0661	1.4360
NdVO_3_	−0.0489	1.3722	YVO_3_	−0.0320	1.2890
ErFeO_3_	−0.0030	1.1867	PuCrO_3_	−0.0450	1.5199

## Data Availability

The data presented in this study are available in the Supplementary Materials.

## Supplementary Materials

The following supporting information can be downloaded at: https://www.mdpi.com/article/10.3390/ma17153741/s1, Table S1: Description of dataset features.
