# Supplementary material for: A Perovskite Material Screening and Performance Study Based on Asymmetric Convolutional Blocks
Source: Materials (Basel). 2024 Jul 28;17(15):3741. doi: 10.3390/ma17153741 (PMC11313385; doi:10.3390/ma17153741)
Supplement: Supplementary file 1 [file materials-17-03741-s001.zip › materials-3114909-supplementary.pdf]

Supplementary Materials: Table S1: Description of dataset features

Name	Unit	Description
formula	None	Chemical formula of the entry
a	Å	Lattice parameter a
b	Å	Lattice parameter b
c	Å	Lattice parameter c
alpha	°	Lattice angle alpha
beta	°	Lattice angle beta
gamma	°	Lattice angle gamma
E_form	eV	Formation energy
E_form oxygen	eV	Formation energy of oxygen vacancy
E_hull	eV/atom	Energy above convex hull, wrt. OQMD db
vpa	A^3/atom	Volume per atom
gap pbe	eV	Bandgap in eV from PBE calculations
r_A	Å	The ionic radius of the element at the A position
r_B	Å	The ionic radius of the element at the B position
t	None	Goldschmidt tolerance factor
μ	None	Octahedral factor
r_A/r_O	None	The ratio of the radius of the A-ion to the oxygen ion
